# DNA Electrochemical Sensor Based on Exonuclease III-Assisted Cycling Signal Amplification for Ultrasensitive Detection of Genetically Modified Soybean

**DOI:** 10.3390/bios16050279

**Published:** 2026-05-11

**Authors:** Lidan Niu, Siyu Huang, Jinmei Zhao, Wenjing Yang, Zhengnan Li, Siqi Niu, Jianchun Yang, Shiqi Chen, Qihui Wang

**Affiliations:** 1 School of Chemistry and Chemical Engineering, Chongqing University, Chongqing 400044, China; niulidan@cqifdc.org.cn (L.N.); huangsiyu@cqifdc.org.cn (S.H.); yangwj308@cqu.edu.cn (W.Y.); 20230650@stu.cqu.edu.cn (Z.L.); 2 Key Laboratory of Condiment Supervision Technology, State Administration for Market Regulation, Chongqing Institute for Food and Drug Control, Chongqing 400713, China; 3 School of Mechanical and Intelligent Manufacturing, Chongqing University of Science and Technology, Chongqing 401331, China; 2023206077@cqust.edu.cn (J.Z.); 2025444001@cqust.edu.cn (S.N.)

**Keywords:** Exo III-assisted target recycling, electrochemical biosensor, CP4-EPSPS gene

## Abstract

The safety of genetically modified crops, particularly the commercial cultivation of glyphosate-resistant genetically modified soybeans, has given rise to significant public concern. Consequently, there is an urgent need to develop efficient and precise methods for detecting genetically modified components. The present study constructed a novel electrochemical biosensor based on nucleic acid exonuclease III (Exo III)-assisted cyclic signal amplification and hairpin probe recognition for the highly sensitive and specific detection of the CP4-EPSPS gene in genetically modified soybeans. The sensor achieves exponential signal amplification by triggering Exo III to cyclically cleave the hairpin probe (H1) upon target DNA binding. Subsequent to this, the released DNA fragments hybridize with the methylene blue-labeled signal probe (HS-MB) that has been immobilized on the electrode surface. This process induces conformational changes and a decrease in the current signal, thereby enabling quantitative analysis of the target gene. The experimental phase of the study successfully validated the sensor’s mechanism and systematically optimized key parameters such as Exo III concentration and reaction time. In optimal conditions, the sensor demonstrated excellent linearity with target DNA concentrations ranging from 100 fM to 10 nM, achieving a detection limit as low as 0.1072 pM. Furthermore, it exhibited remarkable repeatability and stability. This study provides an analytical tool with broad application prospects for the rapid and precise detection of genetically modified crops.

## 1. Introduction

As living standards improve, people are paying increasing attention to food safety. Over the past two decades, the introduction of genetically modified crops has had a profound impact on global agricultural development [1,2,3,4,5]. In the midst of these considerations, the safety of genetically modified foods remains a subject of considerable contention, both in terms of public perception and regulatory oversight. As a vital oilseed and food crop [6,7], soybeans are cultivated worldwide. In order to enhance yields, glyphosate-resistant genetically modified soybeans have been developed and commercialized on a global scale, resulting in a dominant presence within the genetically modified crop market [8]. The 5-enolpyruvylshikimate-3-phosphate synthase (EPSPS) sequence in genetically modified soybeans is derived from the 5-enolpyruvyl-acetyl-pyruvate synthase gene (CP4 gene) and its corresponding protein, synthesized by *Agrobacterium tumefaciens*. The gene in question encodes 5-enolylpyruvyl-3-phosphate (CP4-EPSPS), a glyphosate-resistant compound synthesized by *Agrobacterium tumefaciens* strain CP4, and the corresponding protein. This sequence has been demonstrated to confer a certain level of herbicide tolerance to the crop [9], thereby enabling it to continue growing after emergence without significant adverse metabolic effects [10,11].

As indicated by related studies, while glyphosate is acknowledged to possess a lower degree of toxicity than aspirin [12], experts hold the opinion that it poses no significant hazard to mammals. However, further studies have revealed that glyphosate inhibits detoxifying cytochrome P450 (CYP) enzymes [13]. As demonstrated in the relevant literature, rats chronically exposed to glyphosate from the prenatal period exhibit a significantly increased susceptibility to multiple benign and malignant tumors, including leukemia, skin, liver, thyroid, nervous system, ovary, mammary gland, adrenal glands, kidney, urinary bladder, bone, endocrine pancreas, and hemangiosarcoma [14]. Furthermore, the disruption of gut bacteria in cattle and poultry has been observed [15]. Moreover, extensive research has demonstrated that genetically modified crops pose a significant threat to soil microbial ecology and diversity [16]. The public’s concerns regarding the safety of genetically modified crops remain significant, and in certain regions, their cultivation is prohibited [17]. Consequently, the accurate detection of genes associated with genetically modified crops is imperative.

Bioassays are currently the most commonly used method for detecting genetically modified crops, primarily focusing on the analysis of crop proteins and DNA. The process of protein detection principally involves the binding of target proteins to a colorimetric detection system via specific antibodies [18]. Concurrently, the detection limit of protein immunoassays can predict the presence of recombinant proteins in genetically modified organisms with ≥1% genetically modified content. Concurrently, ultra-high-performance liquid chromatography coupled with tandem mass spectrometry (UHPLC-MS/MS) has garnered significant attention for its ability to simultaneously separate, identify, and quantify thousands of low-abundance proteins in a single run. Consequently, this method has also been employed to assess the safety of genetically modified soybeans containing the EPSPS-related insert [19]. Among the various DNA detection methods, the most advanced technique currently is event-specific qualitative (ES-qual) and quantitative polymerase chain reaction (qPCR). This technology was employed for the qualitative and quantitative analysis of the genetically modified soybean event DP-356043-5 [20]. While these methods can yield detectable results, protein immunoassays remain susceptible to variations in protein expression levels and post-translational modifications. Furthermore, the specificity of antibodies may result in false positives or false negatives. Mass spectrometry-based protein detection offers high throughput; however, the expense of the necessary equipment and the complexity of the associated procedures render this approach impractical for the purpose of rapid on-site screening. Furthermore, while event-specific PCR is both sensitive and specific, it relies on thermal cyclers and fluorescence detection, resulting in time-consuming operations that are susceptible to inhibitor interference. It is evident that prevailing biological detection methodologies are encumbered by their intrinsic methodological technical dependencies and inherent complexity. These methodologies manifest considerable deficiencies with regard to detection throughput, speed, cost, and ease of use. Consequently, there is an urgent need to develop a novel tDNA detection method that is low-cost, highly efficient, and possesses high specificity.

While PCR-based methods (conventional/qPCR) remain the gold standard for GM detection, they require thermal cycling instrumentation, multi-enzyme systems (polymerases, reverse transcriptases), fluorescent probes (for qPCR), and stringent contamination control due to amplicon generation. In contrast, electrochemical biosensors have attracted considerable attention from researchers due to their straightforward preparation and cost-effectiveness. The primary advantage of this technology lies in its integration of biorecognition with physical conduction. These sensors facilitate highly specific and sensitive detection of target signals, obviating the necessity for expensive and complex instrumentation [21,22,23,24,25]. However, existing electrochemical sensors for detecting the CP4-EPSPS are limited in various key performance areas, such as sensitivity, ease of use, cost and detection efficiency. Often, the pursuit of high sensitivity necessitates compromises in terms of operational steps, reagent costs, or reaction duration [26,27,28,29]. To overcome these limitations, introducing efficient and controllable signal amplification strategies is crucial. Exo-III, a key molecular enzyme tool, has attracted significant attention due to its unique enzymatic properties [30,31,32]. Firstly, the enzyme is a double-strand-specific exonuclease that catalyzes stepwise hydrolysis from the 3′-hydroxyl termini of blunt-ended or 3′-recessed double-stranded DNA, while exhibiting negligible activity toward 3′-protruding (3′-overhang) termini Secondly, Exo III exhibits a multitude of enzymatic activities. Beyond its primary exonuclease activity, it exhibits endonuclease activity at adenine-purine (AP) sites, RNase H activity, and 3′-phosphatase activity. These functions endow Exo III with an exceptionally broad range of applications. In conclusion, the process offers the advantages of controllability and mild reaction conditions. During experimentation, precise control over DNA degradation levels can be achieved by simply adjusting reaction temperature and duration, ensuring accurate and reliable experimental outcomes. Collectively, these characteristics endow EXO III with immense potential as an efficient signal amplification component in constructing highly sensitive electrochemical biosensors.

**Figure 1:**
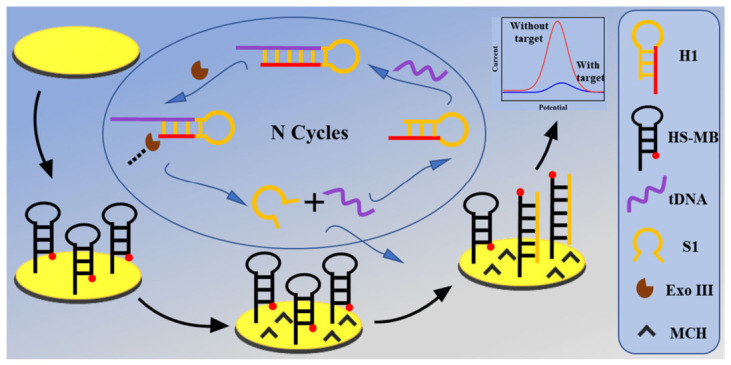
Schematic of a bioelectrochemical sensor for tDNA detection based on Exo III-assisted amplification and hairpin recognition.

Leveraging the outstanding performance of both components, we developed a novel detection strategy that ingeniously couples the precise recognition capability of hairpin probes with the efficient cyclic amplification function of Exo III. This approach enables ultra-sensitive detection of the CP4-EPSPS gene, demonstrating significant application potential. As shown in Figure 1, this detection strategy is based on an Exo III cyclic amplification reaction triggered by target DNA. The process proceeds as follows: First, target DNA hybridizes with the recognition domain of hairpin probe H1, causing H1’s 3′-end to transform from its initial protruding structure into a recessed terminus accessible to Exo III, thereby activating its cleavage activity. Exo III then initiates continuous digestion of H1 from the 3′ end, releasing tDNA and a single-stranded DNA fragment (S1). The released target DNA can rebind to a new H1 probe, initiating a new round of cleavage cycles and enabling exponential amplification of both the target DNA and the single-stranded DNA fragment. Subsequently, the released S1 fragment hybridizes with methylene blue (HS-MB) immobilized on the gold electrode surface via Au-S bonds, unfolding the HS-MB hairpin structure. This conformational change displaces the methylene blue label away from the electrode surface, significantly reducing electron transfer efficiency between the label and electrode. This manifests as a diminished characteristic reduction current of methylene blue, enabling ultrasensitive detection of target DNA based on this signal change.

## 2. Materials and Methods

### 2.1. Materials and Apparatus

Exo III was purchased from Shengong Biotechnology Co., Ltd (Shanghai, China). Other inorganic reagents, including NaCl, Tris-HCl, MgCl_2_, KCl, Na_2_HPO_4_, NaH_2_PO_4_, ammonium persulfate (APS), and tris(2-carboxyethyl)phosphonate hydrochloride, were all of analytical grade and supplied by Aladdin Co., Ltd. (Shanghai, China). 6-Mercapto-1-hexanol (MCH) was supplied by Sigma-Aldrich (Shanghai, China). Ultrapure water (18.2 MΩ·cm, 24 °C) was used to prepare solutions in this experiment; anhydrous ethanol was obtained from Chongqing Chuandong Chemical Co., Ltd (Chongqing, China). A Tris-HCl buffer (pH 7.4) was prepared using 10 mM Tris-HCl, 100 mM NaCl, 10 mM MgCl_2_, and 5 mM KCl.

All DNA used in this study (purified by high-performance liquid chromatography) and DNAmaker were purchased from Shengong Biotechnology Co., Ltd. (Shanghai, China), and their sequences are as follows:

Target: 5′-TTCCAATTTCCATAAACCCCA-3′

H1: 5′-CATAAACCGATCGCTGTGGGGTTTATGGAAATTGGA-3′

HS-MB: 5′-SH-C6- TTCAGCGATCGGTTTA AAGTCGCTG-MB-3′

M1: 5′-TTCCAATTTCCATAAACTCCA-3′

M2: 5′-TTCCAATTTCCATGAACTCCA-3′

M3: 5′-TTCCAATTGCCATGAACTCCA-3′

M23: 5′-TAGCAGTGCAGCTATAGATCA-3′

### 2.2. Preparation of the HS-MB Modified Electrode

Thiol-modified HS-MB was first treated with 10 mM hydrogen trichloride (2-carboxyethyl) phosphate (TCEP) for 1 h in the dark to cleave the disulfide bonds. The reduced HS-MB solution (0.8 μM, containing trichlorohydrate buffer) was subjected to an annealing cycle at 95 °C for 10 min, followed by gradual cooling to 25 °C at 0.5 °C/min.

Gold electrodes (AuE, diameter: 2 mm) were pre-treated according to a previously reported method [33]. In this study, the electrode was immersed in freshly prepared piranha solution (98% H_2_SO_4_: 30% H_2_O_2_, *v*/*v* = 3:1) for at least 30 min (note: the solution reacts violently with organic solvents). The Au electrodes were subject to a series of meticulous polishing procedures, employing alumina slurries with nominal grain sizes of 0.3 µm and 50 nm, respectively. This was followed by a thorough five-minute ultrasonic cleaning process, involving alternation between ultrapure water and ethanol, to ensure optimal cleaning and removal of any residual contaminants. After achieving a clean surface, the electrodes were electrochemically cleaned in 0.5 M H_2_SO_4_ (cyclic voltammetry from −0.3 V to +1.5 V at 100 mV/s until a stable redox peak appeared, confirming complete removal of the oxide). The electrodes were then carefully rinsed and dried with nitrogen.

Dispense 6 µL of the annealed HS-MB solution (1nM) onto the pre-treated AuE. Then, incubate the modified electrode in the dark at 25 °C for 12 h to form the HS-MB/AuE biointerface. Subsequent passivation of the electrode with 1 mM 6-peryl-1-hexanol (MCH) over 2 h resulted in the blocking of non-specific sites, leading to the formation of MCH/HS-MB/AuE.

### 2.3. Assembly of Bioelectrochemical Sensors

Firstly, the individual nucleotide sequences must be diluted using a Tris-HCl buffer at pH 7.4. A reaction mixture (10 μL tDNA, 10 μL H1 (1.5 nM), 10 μL Exo III (1 U/μL)) was incubated at 37 °C for 90 min with agitation, then heated at 80 °C for 10 min to inactivate Exo III. Subsequently, 10 μL of supernatant was added to MCH/HS-MB/AuE and incubated (37 °C, 100 min). After PBS (pH 7.4) rinsing, the biosensor was ready for detection.

### 2.4. Electrochemical Analysis

The electrochemical measurements were conducted on a CHI660E electrochemical workstation (CH Instrument Co., Ltd., Shanghai, China) using a three-electrode system. This comprised a modified gold electrode (2 mm in diameter) as the working electrode, a silver/silver chloride (Ag/AgCl) reference electrode, and a platinum sheet counter electrode. The process of fabricating the electrochemical biosensor was evaluated using electrochemical impedance spectroscopy (EIS) and cyclic voltammetry (CV) in a phosphate-buffered saline (PBS) solution containing 5 mM [Fe(CN)_6_]^3−/4−^ and 0.1 M KCl (pH 7.4). Square-wave voltammetry (SWV) tests were performed in a PBS solution containing 0.1 M KCl (pH 7.4) to characterise redox reaction signals within the potential range of −0.4 to 0 volts (V). The test parameters encompassed a 25 mV amplitude signal, a 4 mV step potential, a 50 Hz frequency, and a scanning range of −0.4 to 0 V (The evaluation findings were baseline-calibrated by utilising the integrated plug-in feature of the CHI660E electrochemical workstation).

### 2.5. Polyacrylamide Gel Electrophoresis Analysis

Polyacrylamide gel electrophoresis (PAGE) was performed in accordance with the following protocol: A 12% polyacrylamide gel was pre-cast. Samples (10 μL) were mixed with 2 μL of 6× loading buffer, and the mixture subsequently injected into the gel lane. Initially, the electrophoresis was performed at a constant current of 10 mA for 5 min, followed by a constant voltage of 120 V for 45 min in 1× TBE buffer. The gel was stained by immersion in Gel-Red for 10 min, and the gel imaging and the subsequent image processing were carried out using the Doc XR+ Bio-Rad Gel Imaging Analysis System (Bio-Rad Laboratories, San Francisco Bay, LA, USA).

## 3. Results

### 3.1. Viability Characterization of Biosensors

**Figure 2:**
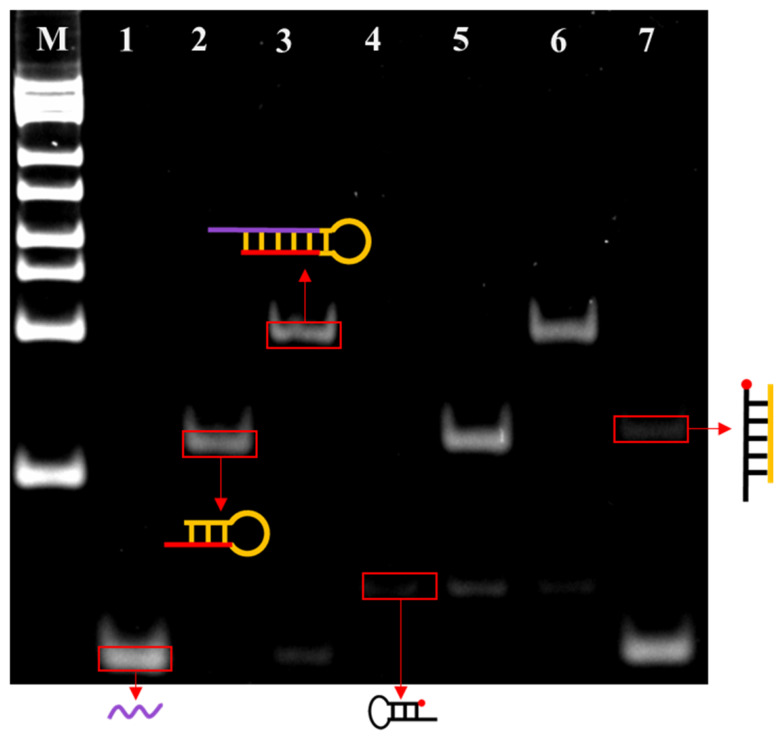
Feasibility characterization in solution. PAGE analysis of Exo III-assisted target recycling. Lane M: DNA marker of 25–500 bp. Lane 1: tDNA; Lane 2: H1; Lane 3: tdna+H1; Lane 4: HS-MB; Lane 5: H1+HS-MB; Lane 6: tDNA+H1+HS-MB; Lane 7: tDNA+H1+Exo III+HS-MB.

The feasibility of this sensor construction strategy was initially validated through the use of polyacrylamide gel electrophoresis (PAGE). As shown in Figure 2, the PAGE characterisation revealed the progression of each reaction step: following hybridization of tDNA with hairpin probe H1, its electrophoretic mobility significantly decreased (lane 3), confirming the successful formation of the tDNA-H1 complex. Following the introduction of Exo III into the system (lane 7), the band that had been observed to migrate slowly disappeared, while the band corresponding to tDNA reappeared. Concurrently, a new, faster-migrating band was observed, which was identified as the complex formed between S1 (the digestion product of H1) and HS-MB. This phenomenon indicates that the target recognition reaction designed by the experimenters has successfully triggered Exo III’s catalytic cleavage of H1, thereby releasing tDNA and S2. Furthermore, the reaction between H1 and HS-MB did not result in any change in their respective migration rates (lane 5). This result clearly demonstrates the capability of S1/S2 to recognise the target ctDNA. Collectively, these results demonstrate that the Exo III-assisted cyclic reaction efficiently releases large quantities of S1. Subsequent to the release of the S1, binding occurs between the signal probe HS-MB, thus achieving a separation of the MB from the electrode surface and, consequently, a substantial reduction in the current signal. This finding serves to validate the operational mechanism of the sensor in question.

### 3.2. Electrochemical Characterization

**Figure 3:**
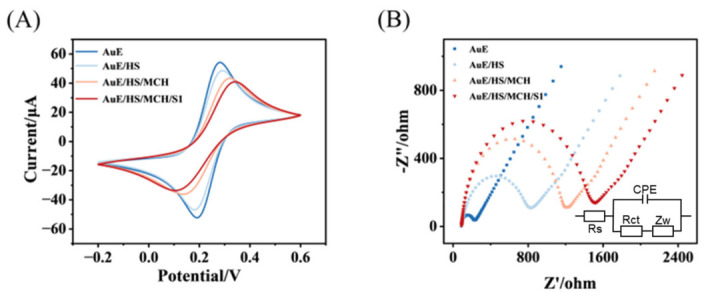
CV (**A**) and EIS (**B**) of different modified electrodes in 0.1 M PBS (pH 7.4) aqueous solution containing 0.1 M KCl and 5.0 mM [Fe(CN)_6_]^3−/4−^. The inset shows the electrical equivalent circuit.

In order to provide further validation of the feasibility of the process under investigation, the stepwise assembly process of the electrochemical sensor was characterized using cyclic voltammetry (CV) and electrochemical impedance spectroscopy (EIS). As demonstrated in Figure 3A, the pristine gold electrode displays distinct redox peaks, thereby confirming its excellent electrochemical performance. Subsequent to the immobilization of DNA and MCH in a sequential manner, a substantial decline in the redox current was detected. This phenomenon can be attributed to electrostatic repulsion between the negatively charged DNA backbone and [Fe(CN)_6_]^3−/4−^, which forces potassium ferricyanide molecules to traverse or come extremely close to this organic molecular layer for electron transfer to occur. This layer has been demonstrated to enhance the effective electron tunnelling distance, consequently leading to a substantial reduction in the electron transfer rate constant, which is characterized by a decline in current. Concurrently, the introduction of MCH results in the occlusion of the remaining active sites on the electrode surface, thereby inducing a further reduction in the redox current. This phenomenon indicates that when S1 binds to the hairpin probe HS-MB modified on the electrode surface, the signal molecule MB originally labeled on HS-MB is subsequently pulled away from the electrode surface. This weakens the electron transfer efficiency between Fc and the electrode, which ultimately manifests as a significant decrease in redox current. This result provides direct electrochemical evidence confirming the specific recognition and binding between S1 and HS-MB. The trend in EIS demonstrated in Figure 3B is consistent with our expectations, exhibiting a highly consistent trend with the CV data. The collective analysis of these results indicates the successful preparation of the biosensor.

### 3.3. Optimization of Experimental Conditions

**Figure 4:**
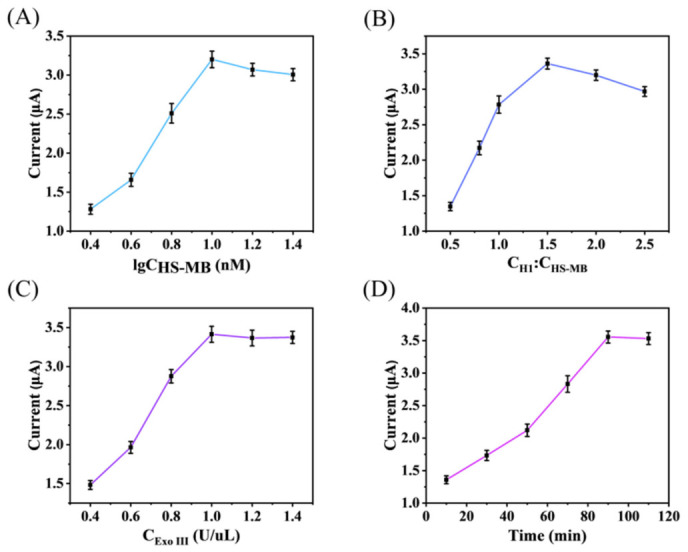
(**A**) Concentration optimization of HS-MB on the electrode; (**B**) Concentration optimization of probe H1; (**C**) Concentration optimization of Exo III; (**D**) Shear time optimization of Exo III. (*n* = 3).

Systematic optimization of experimental conditions is crucial to establish a robust and efficient sensor detection protocol. This process maximizes detection efficiency, minimizes resource consumption and experimental error, enhances the reliability and reproducibility of the results, and provides a solid parameter foundation for subsequent investigations. Consequently, we optimised the following key parameters by analysing the baseline-corrected peak current response of MB using SWV. As shown in Figure 4A, the sensor’s current response increased gradually as the HS-MB concentration rose from 0.4 nM to 1.4 nM. This trend indicates that a higher probe loading on the electrode surface enhances detection sensitivity. However, beyond 1.4 nM, the current response plateaued, suggesting that the available binding sites on the electrode surface became saturated, preventing effective immobilization of additional probes. Therefore, 1.4 nM was chosen as the optimal HS-MB concentration for further experiments. The concentration ratio of H1 to HS-MB is another critical parameter governing reaction efficiency. At a low H1:HS-MB ratio, the limited release of S1 fails to activate all immobilized HS-MB probes, leading to a suboptimal signal. Conversely, an excessively high ratio introduces steric hindrance due to an overcrowding of HS-MB molecules on the electrode surface. This hindrance impedes efficient S1 binding and probe activation, ultimately attenuating the signal amplification. Figure 4B shows that the current signal amplitude increased with the H1:HS-MB ratio from 0.5 to 1.5, reaching a maximum at a ratio of 1.5. Further increase in the ratio led to a signal decline, confirming that steric hindrance becomes the limiting factor. Therefore, an H1:HS-MB molar ratio of 1.5 was determined to be optimal for achieving maximum recognition and conversion efficiency in the reaction system.

The concentration of Exo III is a critical parameter for sensor performance, as it directly determines the abundance of active signaling molecules generated. Therefore, we first optimized the Exo III concentration. As shown in Figure 4C, the peak current increased with Exo III concentration and reached a plateau at 1 U/μL, which was consequently selected as the optimal concentration. Furthermore, the Exo III-assisted cyclic amplification involves both target recognition and enzymatic digestion. An insufficient reaction time may lead to incomplete processes, whereas an excessively long duration is inefficient. Thus, we also optimized the digestion time. Figure 4D shows that the current signal increased with time and began to stabilize at approximately 90 min, which was therefore chosen as the optimal reaction time.

These optimizations establish a solid experimental foundation for reliable sensor detection in complex samples and provide a parameter framework for constructing similar biosensing systems.

### 3.4. Performance of Electrochemical Sensors

**Figure 5:**
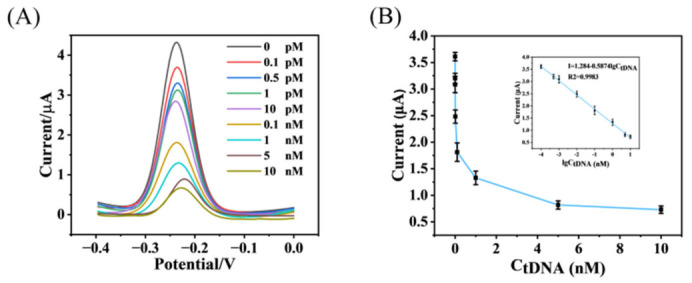
(**A**) SWV curves of tDNA at different concentrations (**B**) Peak current signals of tDNA at different concentrations and their calibration curves (*n*  =  3).

In order to evaluate the performance of the ratiometric electrochemical sensor under the reaction conditions that had been optimised, the sensor was exposed to ctDNA at concentrations ranging from 10 nM to 0.1 pM. Figure 5A displays the wave-voltage curves of this electrochemical sensor under different tDNA concentrations. As the tDNA concentration increases, the peak current signal exhibits a significant decreasing trend. This phenomenon indicates that increased tDNA concentration in the system promotes greater hybridization between S1 and HS-MB immobilized on the electrode surface. This hybridization causes the stem-loop structure of HS-MB to open, thereby distancing the MB labeled at its terminal from the electrode surface. This conformational change increases the spatial distance for electron transfer, reducing the efficiency of electron transfer between the electrode and the electroactive substance in the solution, ultimately leading to a decrease in the current signal. Calculations revealed a good linear relationship between the peak current signal and the logarithm of tDNA concentration from 0.1 pM to 10 nM, with the linear equation I = 1.284 − 0.5874lg*c*_CtDNA_ and a correlation coefficient of 0.9983, indicating an excellent positive linear relationship between the two variables (Figure 5B). Using the 3σ/k method, the detection limit of this approach was calculated to be 0.1072 pM, demonstrating its capability to detect extremely low concentrations of tDNA. In comparison to the other CP4-EPSPS detection assays summarized in Table 1, the proposed electrochemical approach demonstrates a superior limit of detection (LOD) and surpasses the performance of most state-of-the-art technologies. These findings underscore its great promise for the accurate and reliable detection of trace CP4-EPSPS targets. These results fully demonstrate the strong application potential of our developed bioelectrochemical sensor for high-precision, high-reliability detection of low-concentration tDNA, providing robust support for real-time monitoring and risk assessment.

**Figure 6:**
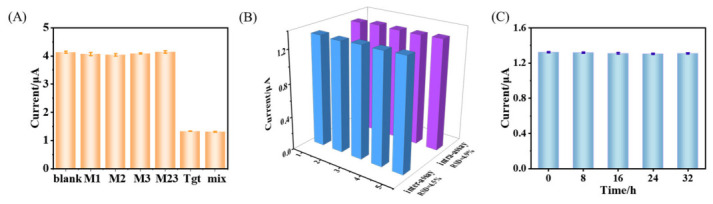
(**A**) Selectivity investigation of target tDNA challenging mismatched sequences. Samples include blank control, M1-DNA (1 nM), M2-DNA (1 nM), M3-DNA (1 nM), M23-DNA (1 nM), tDNA (1 nM) and mixtures containing the above analog interferences (10 nM each). (**B**) Inter- and intra-assay reproducibility of biosensors with 100 pM tDNA. (**C**) Storage stability of the biosensor incubated with 1 nM tDNA (*n*  =  3).

Evaluating the specificity of innovative biosensors is paramount, as specificity serves as the decisive factor for the successful construction of target-detecting sensors. In this study, a series of interfering sequences at 1 nM concentration were analyzed, including single-base mismatched (designated M1), double-base mismatched (M2), triple-base mismatched (M3), and fully mismatched (M23) variants, whose sequences are all listed in Section 2.1. The responses of these variants were then compared with the target sequence. As displayed in Figure 6A, the sensor exhibits a distinct signal change exclusively in the presence of the correct target analyte. These results demonstrate that the proposed method possesses excellent target specificity, effectively distinguishing the target sequence from similar non-target sequences, including those with single-base mismatches. The enhanced discrimination capability of the assay is of paramount importance, as it significantly reduces the risk of both false-positive and false-negative responses. Its exceptional specificity primarily stems from HS-MB’s precise recognition capability of S1.

As shown in Figure 6B, five electrodes were prepared in one batch and five in another, under the same conditions. Their ratiometric signal also exhibited excellent intra- and inter-assay reproducibility, with relative standard deviations (RSDs) of 4.5% and 4.9%, respectively. As demonstrated in Figure 6C, for the prepared electrochemical sensors, the signal ratio remained stable after 8, 16, 24, and 32 consecutive days of storage at 4 °C, suggesting that the sensor possesses excellent stability.

## 4. Conclusions

This experiment successfully constructed a novel bioelectrochemical sensor that achieves ultra-sensitive detection of the CP4-EPSPS gene by coupling the precise recognition capability of hairpin probes with the efficient cyclic signal amplification function of Exo III. In the detection mechanism, Exo III serves as a signal amplification tool. Its activity is triggered by the hybridization of tDNA with hairpin probe H1, thereby enabling a single tDNA molecule to mediate the generation of a large number of S1. The generated S1 fragments further hybridize with HS-MB immobilized on the electrode surface. This hybridization induces a conformational change in HS-MB, causing MB molecules to detach from the electrode surface. This effectively inhibits interfacial electron transfer, generating detectable changes in the current signal, ultimately enabling quantitative analysis of tDNA. In summary, this sensor combines the high specificity of hairpin probes with the cyclization amplification advantage of Exo III, demonstrating outstanding detection sensitivity and specificity. Furthermore, the sensor involves fewer types of nucleic acid sequences, simplifying the construction process and significantly reducing preparation costs. Therefore, the electrochemical sensing strategy proposed in this study shows broad application potential in the field of tDNA detection and holds promise as an effective tool for analyzing tDNA in complex biological samples.

## Figures and Tables

**Table 1:** Comparison of this method with other reported biosensing systems for CP4-EPSPS detection.

Analytical Method	LOD (M)	Reference
SPR	2.5 × 10^−9^	[34]
QCM	4.0 × 10^−9^	[35]
FL	2.0 × 10^−9^	[36]
ECL	2.0 × 10^−10^	[37]
RT-PCR	4.9 × 10^−10^	[38]
FRET	3.0 × 10^−11^	[39]
PEC	5.0 × 10^−11^	[40]
This work	0.1072 × 10^−12^	This work

## Data Availability

The data that support the findings of this study are available from the corresponding authors upon reasonable request.
